# Comparative in vitro study regarding the biocompatibility of titanium-base composites infiltrated with hydroxyapatite or silicatitanate

**DOI:** 10.1186/1754-1611-8-14

**Published:** 2014-06-19

**Authors:** Ioana-Carmen Brie, Olga Soritau, Noemi Dirzu, Cristian Berce, Adriana Vulpoi, Catalin Popa, Milica Todea, Simion Simon, Maria Perde-Schrepler, Piroska Virag, Otilia Barbos, Gabriela Chereches, Petru Berce, Valentin Cernea

**Affiliations:** 1 The Institute of Oncology “Prof. Dr. I. Chiricuta” Cluj-Napoca, Cluj-Napoca, Romania; 2 University of Medicine and Pharmacy “Iuliu Hatieganu” Cluj-Napoca, Cluj-Napoca, Romania; 3 Technical University, Cluj-Napoca, Romania; 4 Faculty of Physics & Institute of Interdisciplinary Research in Bio-Nano-Sciences, Babes Bolyai University, 400084 Cluj-Napoca, Romania

**Keywords:** Implants, Porous titanium, Hydroxyapatite, Silicatitanate, Osteoblasts, Cell adhesion, Differentiation, Mineralization

## Abstract

**Background:**

The development of novel biomaterials able to control cell activities and direct their fate is warranted for engineering functional bone tissues. Adding bioactive materials can improve new bone formation and better osseointegration. Three types of titanium (Ti) implants were tested for *in vitro* biocompatibility in this comparative study: Ti6Al7Nb implants with 25% total porosity used as controls, implants infiltrated using a sol–gel method with hydroxyapatite (Ti HA) and silicatitanate (Ti SiO_2_). The behavior of human osteoblasts was observed in terms of adhesion, cell growth and differentiation.

**Results:**

The two coating methods have provided different morphological and chemical properties (SEM and EDX analysis). Cell attachment in the first hour was slower on the Ti HA scaffolds when compared to Ti SiO_2_ and porous uncoated Ti implants. The Alamar blue test and the assessment of total protein content uncovered a peak of metabolic activity at day 8–9 with an advantage for Ti SiO_2_ implants. Osteoblast differentiation and *de novo* mineralization, evaluated by osteopontin (OP) expression (ELISA and immnocytochemistry), alkaline phosphatase (ALP) activity, calcium deposition (alizarin red), collagen synthesis (SIRCOL test and immnocytochemical staining) and osteocalcin (OC) expression, highlighted the higher osteoconductive ability of Ti HA implants. Higher soluble collagen levels were found for cells cultured in simple osteogenic differentiation medium on control Ti and Ti SiO_2_ implants. Osteocalcin (OC), a marker of terminal osteoblastic differentiation, was most strongly expressed in osteoblasts cultivated on Ti SiO_2_ implants.

**Conclusions:**

The behavior of osteoblasts depends on the type of implant and culture conditions. Ti SiO_2_ scaffolds sustain osteoblast adhesion and promote differentiation with increased collagen and non-collagenic proteins (OP and OC) production. Ti HA implants have a lower ability to induce cell adhesion and proliferation but an increased capacity to induce early mineralization. Addition of growth factors BMP-2 and TGFβ1 in differentiation medium did not improve the mineralization process. Both types of infiltrates have their advantages and limitations, which can be exploited depending on local conditions of bone lesions that have to be repaired. These limitations can also be offset through methods of functionalization with biomolecules involved in osteogenesis.

## Background

Current procedures to repair bone defects include the use of grafts or implants, yet these approaches face important limitations. Bone tissue engineering has seen tremendous development over the years, as much collaborative effort of scientists, surgeons and engineers has been spent to create bone implants and/or grafts which enhance bone repair and regeneration.

The very first requirements of any implantable material are related to the toxicological aspects: bone implants/grafts need to be sterile, free of pyrogens, made of pure molecules and biocompatible with tissues and body fluids [1]. The classic paradigm of bone tissue engineering highlights several key players: a biocompatible scaffold, osteogenic cells, morphogenic signals and vascularization [2]. The biocompatibility requirement stresses the selection of bone graft/implant composition so that it could be accepted and integrated by the host tissue.

The biomaterial’s surface topography, chemistry and mechanical characteristics influence cell functions by triggering specific molecular events at the cell-material interface: cell adhesion, spreading, migration, proliferation and differentiation [3-5].

One of the best biomaterials is titanium (Ti), which stands out with its remarkable properties: good biocompatibility, low density, good mechanical properties and better resistance to corrosion. But, even if well tolerated by the human body, Ti and its alloys are bioinert and cannot promote tissue bonding to the implant. Many clinical problems regarding Ti implants are reported in the literature, such as weakness of osseointegration in the case of long-term implants and tissue inflammatory response at the implantation sites due by release of titanium particles in surrounding tissues [6-8]. Other problems are caused by loss of ability of bone remodeling caused by implant stiffness and biomechanical mismatch [9,10].

In order to solve those medical problems, surface coating of titanium implants with specifically engineered *bioactive materials* is beneficial in that it stimulates new bone formation and promotes better osseointegration [11]. *Bioactivity* is a surface property of the implant which allows the chemical integration of synthetic materials with the host’s tissue, inducing the formation of extracellular matrix with biomineralization of calcium phosphate nanocrystallites at the bioactive substrate/tissue interface [12]. New methods that combine the bioactivity of HA or bioactive glass and the mechanical properties of Ti or Ti alloys have been intensively investigated in the past decades, and implants coated with plasma-sprayed HA have already entered the clinical practice [13,14]. The chemical and crystallographic structure of HA is similar to bone minerals and consequently is biocompatible and osseoconductive, yet its poor mechanical properties are obstacles in the designing of bone implants [15]. The release of toxic elements by the metal implants coated with bioactive ceramics and the differences in thermal expansion between the ceramic substrate and metal are other disadvantages [16]. In order to avoid brittleness and to increase the bond strength between HA and Ti alloys, different methods of HA coatings were tested: plasma spray, pulse laser-deposition [17,18], combined laser and induction plasma spraying [19], mechanical alloying [20], sol–gel process [21], HA growth in simulated body fluid [22] or electrophoretic deposition [23]. A method for obtaining biocomposites from Ti powder, HA and bioactive glass, with the aim of improving the mechanical and biological properties of HA, was described by Ning et al. [15].

Bioactive glasses coatings of metal implants are also used to improve bone-binding ability by promoting protein adsorbtion and forming biologically active apatite layers upon implantation [24,14]. Saino et al. reported enhancement of human osteoblasts SAOS-2 calcium deposition after culturing on Ti-6Al-4 V scaffolds coated with bioglass [25].

In the present study, the *in vitro* biocompatibility was determined using Ti6Al7Nb implants with 25% total porosity, processed with Selective Laser Melting (SLM) technology, infiltrated with hydroxyapatite and silicatitanate using a sol–gel method, in an attempt to improve the bioactivity of the material. Human osteoblast behavior was observed in terms of adhesion, cell growth and differentiation. The ability of Ti implants to induce osseoinduction was studied by scanning electron microscopy (SEM) and fluorescence microscopy with cytochemical stains for cell adhesion. Osteoblast proliferation was assessed through viability tests and assessment of total protein synthesis, whilst the expression of molecules involved in osteoblast differentiation (osteopontin, osteocalcin, alkaline phosphase and collagen) was investigated through immunocytochemical staining and quantitative assays. The mineralization process, as an important component of implant integration in bone tissue, was evaluated through measurements of the calcium deposits on the Ti implants. The experiments were conducted under different environmental conditions: standard medium with fetal calf serum (FCS), serum-free medium, specific osteogenic differentiation mediums: simple and complex (supplemented with growth factors).

## Materials and methods

### Implants

The atomized Ti6Al7Nb powder (MCP HEK GmbH), medical grade, as the control material, was processed with a Selective Laser Melting machine Realizer II SLM 250 with a Nd: YAG (fibre laser). Test specimens of 10 mm × 5 mm × 3 mm were manufactured with a laser power of 50 W, the laser spot size 150 μm, layer thickness 50 μm, hatch spacing 100 μm and scan speed 400 mm/s. The successive layers were deposited in the “z” direction. The samples were built on a titanium plate whose temperature was kept at approximately 473°K (200°C), in argon atmosphere. The manufactured specimens were cleaned ultrasonically in distilled water for 10 min. and dried at 353°K (80°C) for 30 min [26].

In order to improve the bioactivity of the resulted titanium base specimens, their pores were infiltrated with calcium phosphate precipitates and silicatitanate gel, respectively. Two types of calcium phosphate precipitates A (pH = 4.5) and B (pH = 10) were synthetized through wet chemical precipitation from calcium nitrate tetrahydrate-Ca (NO_3_)_2_•4H2O and diammonium hydrogen phosphate-(NH_4_)2HPO_4_ (Sigma Aldrich). The silicatitanate gel was obtained by the sol–gel method from titanium isopropoxide (TIP) with the formula Ti{OCH(CH_3_)_2_}_4_ and tetraethylorthosilicate (TEOS) with the formula Si(OC_2_H_5_)_4_. The preparation of TiO_2_/SiO_2_ gel included 2 steps. The first step involved the hydrolysis of Ti{OCH(CH_3_)_2_}_4_ to form the uniform sol. TIP was diluted with ethanol and a small amount of nitric acid HNO_3_ to form a transparent colloid. Then, distilled water with a small amount of nitric acid and the rest of ethanol were mixed together and dropped into the above colloid solution. The molar ratio of Ti{OCH(CH_3_)_2_}_4_:C2H5OH:H2O:HCl was 1:15:10:0.89. The colloid was then left to homogenize at room temperature for one hour. In the second step, TEOS was hydrolysed with a molar ratio of Si(OC_2_H_5_)_4_:C2H5OH:H2O:HCl = 1:7.6:25:0.28. The TiO_2_ solution was then added dropwise to the above SiO_2_ sol to form the transparent TiO_2_/SiO_2_ sol mixture. The transparent mixture was left to homogenize at room temperature for 30 min before carrying out the infiltration into the pores of the titanium base specimens.

Concerning the infiltration of porous titanium implants, the obtained disks were immersed in the hydroxyapatite (HA) solution and silicatitanate gel, kept in vacuum (100 mbar) for 15 min. Subsequently, the samples were dried at 60°C , then at 110°C in an oven. All the specimens were sintered for 1 h in air atmosphere, using a furnace, at 600°C for samples with HA and 400°C for those infiltrated with silicatitanate gel [20]. The infiltrated samples were sterilized by exposing them to dry heat, at 180°C for 2 hours.

### Cell culture

Human osteoblasts were isolated from patella bone pieces harvested during arthroplasty as described by Tomuleasa et al. [27]. Briefly, the bone fragments were first mechanically processed and then digested for 30 min with an enzymatic cocktail: 0.1% colagenase IV (Gibco) + 0.25% trypsin EDTA-4 (Sigma). The resulted bone explants were cultured in Dulbecco’s modified Eagle’s medium (DMEM)/F-12HAM (Sigma) containing 20% fetal calf serum (FCS), 2 mM L-Glutamine, 1% antibiotics, 1% non-essential aminoacids (NEA) (all reagents from Sigma), in 25-cm^2^ culture flasks (Nunc) in a humidified 7% CO2 atmosphere. The first cells appeared near the explants after 14 days and the culture reached confluence after 6–8 weeks, when the explants were removed and cells were trypsinized and replated. The isolated cells were characterized at the second passage by immunocytochemical staining, showing positivity for osteoblastic lineage markers such as osteonectin (ON) and osteopontin (OP). The obtained cells showed a spindle-like shape in the first passages and a polygonal shape with multiple dendritic extensions in more advanced passages. The medium was changed twice each week and the cells were used after four to six passages.

In the differentiation experiments we used serum-free simple osteogenic medium (OS) consisting on DMEM/F-12HAM without phenol red, 2 mM L-Glutamine, 1% antibiotics, 1% NEA, 10 nM dexamethasone, 50 μg/ml ascorbic acid and 10 mM β-glycero-phosphate or complex osteogenic medium (OC) consisting of the above mentioned simple osteogenic medium but supplemented with growth factors: 3 ng/ml transforming growth factor β1 (TGFβ1) and 4 ng/ml bone morphogenetic protein 2 (BMP2). All the reagents were purchased from Sigma Aldrich. In some experiments cells were grown in the absence of fetal serum (FCS) in DMEM/F12 HAM medium without phenol red (serum-free medium, SF) after day 18, considering that the cell environment is an important component of variability in experiments and that the proteins present in fetal serum (FCS) may interfere with certain tests (e.g. determination of collagen or other proteins).

### Scanning electron microscopy (SEM)

The scaffolds seeded with osteoblasts were fixed with 4% paraformaldehyde in phosphate buffered saline (PBS) after 1 hour and 28 days of cultivation on Ti implants. Implants were washed three times with PBS and immersed in PBS before analysis. Specimens were characterized with a Quanta 3D FEG Scanning Electron Microscope equipped with an energy-dispersive X-ray microanalyzer (EDX). Control implants without cells were also used for SEM and EDX analysis.

### Cell adhesion and proliferation

Cell adhesion at 2 hours and cell proliferation were assessed with the Alamar blue test, which is used mainly to measure cell viability. Resazurine, a non-fluorescent dye is converted to resorufin (red fluorescence) in metabolically active cells, through a reduction mechanism. Fluorescence intensity depends on the number of viable cells. 8 × 10^5^ cells/well were suspended in 1.5 ml DMEM/F12HAM complete medium and seeded on the implants placed in 12-well plates. Each assay was performed in triplicate. After 2 hours of cultivation, 150 μl of Alamar blue (Invitrogen) were added to each well. The plates were incubated for 1 hour at 37°C, in the dark. The medium was subsequently transferred to another 12-well plate and fluorescence intensity was measured using a BioTek Synergy 2 plate reader (excitation 540 nm, emission 620 nm). The cells were quantified at different time intervals: 2 hours, 24 hours, 9 days and 18 days.

Cell adhesion was also assessed through fluorescence microscopy with cytochemical stains: DAPI (4,6-diamidino-2-phenylindole) for nuclei and TRITC (tetramethylrhodamine isothiocyanate) phalloidin (Sigma) for actin filaments. After 1 hour cultivation in complete medium on the Ti implants, cells were fixed with 4% paraformaldehyde solution and permeabilized with 0.1% Triton X-100 in PBS for 20 min at room temperature. TRITC phalloidine (1:20 in PBS) was used for the staining of filamentous actin. The samples were counterstained with an antifade medium containing DAPI (UltraCruz™ Mounting medium-Santa Cruz Biotechnologies) in order to highlight the nuclei. Slides were examined in reversed phase fluorescence with a Zeiss Axiovert D1 microscope, using filters at 340/360 nm for DAPI and 546 nm for TRITC. Cultures of osteoblasts on plastic dishes were used as negative controls. Manufactured discs of synthetic resins composed by 70% wt Bis-GMA(2,2-bis[4-(2-hydroxy-3-methacryloxypropoxy)phenyl]-propane), 30% wt TEGDMA (triethylene glycol dimethylacrylate), 1%DHEPT(Dihydroxyethyl-P*-*Toluidine), 0.035%BHT (Butyl hydroxytoluene), 1.08% BPO (Benzoyl peroxide) and 25.93% βTCP (Tricalcium phosphate) were used as positive control in adhesion studies.

**Protein synthesis**, as an indicator of cell proliferation, was evaluated by determining the total protein content in culture medium and cell lysates, using the microplate BioRad Protein assay - a colorimetric assay based on the Bradford dye-binding method. The test consists in obtaining a calibration curve of standard proteins (bovine serum albumine, BSA), which will serve for extrapolation of the values obtained from samples in spectroscopy absorbance. The osteoblasts were cultured on titanium implants, in 24-well plates, either in complete medium (with FCS) or in serum-free medium. Ten microliters of medium were collected from the cultures at different time points. Cell lysis was performed with CelLyticTM MTMammalian Tissue Lysis/Extraction ReagentCell (Sigma). Ten microliters of samples as well as decreasing known concentrations of standard BSA were added in each well of a 96-well microplate. After adding 200 μl Dye Reagent (diluted 1:4 with deionizated water), the microplate was incubated 15 min at room temperature, and absorbance was measured with a BioTek Synergy 2 microplate reader at 595 nm.

### Cell differentiation and secretion of the extracellular matrix

***Osteopontin (OP)*** was evaluated in cell culture medium (complete medium and serum-free medium) and cell lysates, using a R&D Quantikine ELISA kit according to the manufacturer’s instructions. Briefly, complete medium was harvested at days 8, 14, 21 and 28 and serum-free medium at day 21 and 28. Cell lysates were harvested after 8, 14 and 24 days of cultivation. In each well of a microplate (coated with mouse monoclonal antibody against OP), 50 μl of standards and undiluted samples were added. After 2 hours of incubation at room temperature and washing, 200 μl of OP conjugate were added, followed by 2 hours incubation and further washing. After 30 min incubation with the substrate solution, the stop solution was added and optical density was determined with a microplate Biotek Synergy2 reader set to 450 nm.

***Alkaline phosphatase (ALP) activity*** was assessed in cell medium and cell lysates, using a fluorimetric Alkaline Phosphatase detection kit (Sigma Aldrich). The culture conditions were: medium with FCS, serum-free medium, simple medium and complex differentiation medium. Samples were harvested at different intervals, from 8 to 35 days. 20 μl from each sample, negative control (DMEM/F12 HAM medium without phenol red) and positive control were added in each well of a black 96-well microplate. The samples were incubated for 20 min at 65°C, cooled for 2 min on ice to stabilize at room temperature and 180 μl mixture of Dilution Buffer and Fluorescent Assay Buffer at 1:8 ratio were added. Activity of alkaline phosphatase was measured at different points in time, with a Biotek Synergy 2 fluorometer at 360 nm excitation and 440 nm emission.

***Calcium deposits*** that are formed after the mineralization process can be highlighted with Alizarin red - an anthraquinone derivate and calcium chelator. Osteoblasts seeded and cultivated on titanium implants, either in medium with FCS or serum-free medium, were fixed with 4% paraformaldehyde after 14, 21 and 28 days. After washing with deionized water, Ti implants were incubated for 10 min at room temperature with 2% Alizarin red solution (pH 4.1). After intensive washing of implants a destaining method was used, with 10% (w/v) cetylpyridinium chloride (CPC) (Sigma) in 10 mM sodium phosphate (pH 7.0). After 15 min at room temperature, aliquots from the extracted stain were transferred to a 96-well plate and diluted 10 fold with CPC solution. The violet-colored supernatant was read with a microplate reader at 562 nm.

### Collagen detection

The most abundant protein of the extracellular matrix, collagen, was determined with the SIRCOL collagen assay, a colorimetric method based on staining of collagen with Sirius red. Samples were cultured in serum-free medium, simple medium and complex osteogenic differentiation medium. Medium was harvested at day 21 and 28 for serum-free medium and at day 35 for differentiation medium. 100 μl of collagen standards, samples, negative controls (medium) and 1 ml Sircol Dye Reagent were added to low protein binding microcentrifuge tubes and incubated 30 min on an orbital shaker. After centrifuging and draining the tubes, 750 μl of ice-cold Acid-Salt Wash Reagent were added followed by another centrifugation for removal of the unbound dye. 250 μl of Alkali Reagent were used to release the Sircol dye from the collagen-dye complex. Samples were transferred on a 96-well plate and absorbance was measured with a microplate reader at 555 nm.

### Immunocytochemical staining

Osteoblasts cultivated on titanium implants for 3, 14, 21 and 28 days in complete medium with FCS, were fixed with 4% paraformaldehyde solution and permeabilized with 0.1% Triton X-100 for 20 min at room temperature. The samples were kept for 20 minutes at room temperature with 10% BSA to avoid non-specific antibody binding. Osteopontin, osteocalcine, and collagen 1A1 (all mouse anti-human primary antibodies from Santa Cruz Biotechnologies) were diluted at a ratio of 1:50 in 1% BSA and incubated overnight at 4°C with the samples. Secondary goat anti-mouse antibodies IgG1 marked with FITC (fluorescein isothiocyanate) and Texas Red (Santa Cruz Biotechnologies) were added and incubated for 45–60 minutes at 37°C. Samples were counterstained with an antifade medium containing DAPI in order to highlight the nuclei and were subsequently examined with a reversed phase epifluorescence Zeiss Axiovert D1 microscope at 488 nm for FITC, 546 nm for Texas red and 340/360 nm for DAPI.

### Statistical analysis

Statistical analysis was performed with the GraphPad Prism 5 software, using the Bonferroni Multiple Comparison test. Statistical significance was set at p < 0.05.

## Results

The success of bone grafting with Ti implants depends on the properties of the implant (mechanical features, surface structure, chemical and physical features, porosity) and on the host’s characteristics (bone bed, associated diseases, etc.) [28]. The biochemical and biophysical signals induced at the cell-material interface, which relay complex information resulting from molecular, topographic and mechanical properties of the substrate, trigger a cross-talk between the material and the cells with consequences on cellfunctions such as: early attachment (in the first seconds), cell adhesion and spreading, proliferation and differentiation [21]. The ability of the biomaterial to promote cell adhesion and proliferation is a critical factor for osseointegration. Adhesion allows the host’s osteoblastics to attach to the substrate and activates a complex machinery of reorganization of intra and extracellular molecules. It promotes a specific cellular response consisting of the induction of a differentiation processes towards more specialized cell types, migration, proliferation and gene expression of specific proteins [29]. To enhance bone ingrowth, solutions to enhance surface modification are currently being studied. One of the most promising is *bioactive coating* which seems to accelerate new bone formation at the bone–implant interface [30].

In the present study we used an original method to coat the porous titanium implants, through infiltration with calcium phosphate precipitates and silicatitanate gel, in vacuum conditions followed by sintering at 600°C (for samples with HA) and at 400°C (for silicatitanate samples). The total porosity of Ti control implants before infiltration was evaluated by the Archimedes method (ISO 2738–99) and reached values of nearly 25%. After infiltration of implants total porosity decreased to 22% for titanium infiltrated with hydroxiapatite and to 23% for titanium infiltrated with silicatitanate. The microstructure was analyzed with a metallographic microscope that revealed mainly irregular interconnected pores, with a minimum diameter d_min_ = 70–100 μm and a maximum diameter d_max_ = 200–400 μm [26]. Three different porous titanium implants were obtained and studied: porous titanium without infiltration (Ti Ctrl), titanium infiltrated with hydroxiapatite (Ti HA) and titanium infiltrated with silicatitanate (Ti SiO_2_).

### Structural analysis

The surfaces of the resulted implants were characterized using scanning electon microscopy (SEM) and elemental analysis by energy dispersive X-ray spectroscopy (EDX).

**Figure 1:**
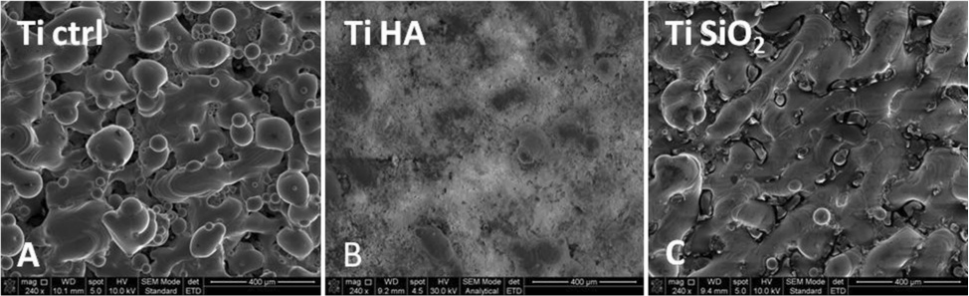
**SEM images of the surfaces of the studied substrate.** Legend **A**. Non-infiltrated titanium, Ti Ctrl; **B**. Titanium infiltrated with hydroxyapatite, Ti HA; **C**. Titanium infiltrated with silicatitanate, Ti SiO_2_ (magnification 240×).

The ***SEM analysis*** revealed differences in the surface structure for the three implants studied (Figure 1). Infiltration with HA yielded a rough surface consisting of HA crystals covering almost entirely the titanium surface (Figure 1B). In case of the silicatitanate-infiltrated titanium (Ti SiO_2_), the pores were covered with silicatitanate gel and the film coating was transparent and almost continuous (Figure 1C).

The ***EDX analysis*** showed a pronounced decrease in titanium elemental content on the surface of HA infiltrated samples (30.25%), and a much lower decrease in Ti SiO2 implants (43.65%). The Ti implants infiltrated with HA had a Calcium (Ca) content of 17.04% and a Phosphorus (P) content of 7.37%, with a Ca/P ratio of 2.31 (Table 1).

**Table 1:** The chemical elemental content of the studied samples (EDX analysis)

**Chemical element**	**Ti control (A%)**	**Ti infiltrated with HA (A%)**	**Ti infiltrated with SiO2 (A%)**
**Aluminum**	9.65	1.42	4.46
**Niobium**	3.37	0.78	1.43
**Titanium**	86.98	30.25	43.65
**Phosphorus**		7.37	
**Calcium**		17.04	
**Silicon**			6.99

Legend: HA- hydroxyapatite, SiO2- silicatitanate.

Most studies in the literature reported Ca/P ratios ranging between 0.5 and 1.9 [31]. *Kitsugi et al.*[32] used four kinds of calcium phosphate ceramics with Ca/P ratios of 1.0, 1.5, 1.66 and 2. Transmission electron microscopy analysis showed that the bone-bonding behavior of calcium phosphate ceramics did not vary with the calcium/phosphate molar ratio [32]. Silica content was 6.99%.

### Osseoinduction: cell adhesion and proliferation

The adherence of cells to the materials was evaluated through SEM imaging and cytochemical staining of nuclei at 1 hour after seeding the cells on titanium implants. Cell proliferation on substrates was assessed by a viability assay (Alamar blue test) and by quantification of the total protein contents in the cell culture medium or cellular lysates.

**Figure 2:**
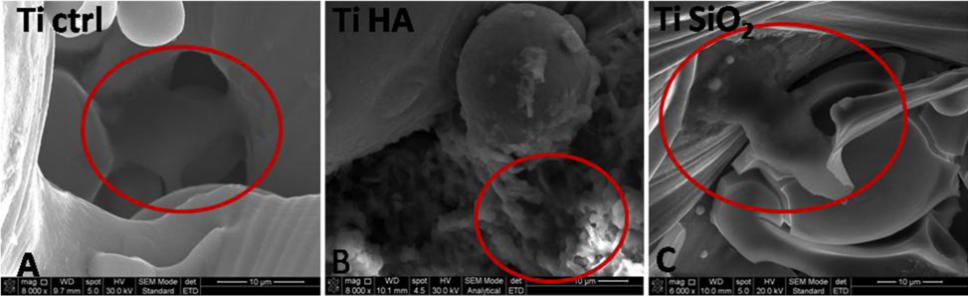
**SEM images of the of studied substrates 1 hour after seeding with osteoblasts, showing cells attached to the surface of the implants.** Legend: **A**. non-infiltrated titanium, Ctrl Ti (magnification 8000×); **B**. titanium infiltrated with hydroxyapatite, Ti HA (magnification 8000×); **C**. titanium infiltrated with silicatitanate, Ti SiO_2_ (magnification 6000×).

**Figure 3:**
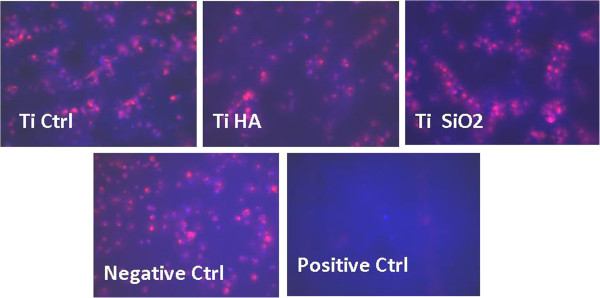
**Fluorescence images (magnification 100×) of osteoblasts attached to the surface of the studied implants.** Legend: cells cultivated on plastic dihes-negative Ctrl, non-infiltrated titanium Ti Ctrl, titanium infiltrated with hydroxyapatite Ti HA and titanium infiltrated with silicatitanate Ti SiO2, composite resins as positive Ctrl. Staining was made with DAPI (for nuclei, blue staining) and TRITC-phalloidin (for actin F, red staining).

SEM images were captured one hour after seeding the implants with osteoblasts (Figure 2). We noticed changes of cell shape, with development of dendritic extensions and their attachment at several points, for the Ti Ctrl (Figure 2A) and Ti SiO2 implants (Figure 2C). Cells grown on Ti HA implants maintained a rounded shape (Figure 2B). Our observations are in agreement with certain data from the literature. A similar behavior of the biomimetic apatite that restricted spreading and promoted extension of cellular projections of MC3T3-E1 preosteoblasts, along the textured surfaces, was observed through confocal microscopy by Chou et al. [33]. In a comparative study of the attachment of osteoblastic cells on titanium and hydroxyapatite, Goto et al. [34] found more osteoblasts attached on HA surface which did not form well-defined and polarized stress fibers or vinculin-positive focal adhesions (as did those cultivated on Ti surface).A quantitative morphometric analysis of cell adhesion was also performed on cells stained with DAPI (for nuclei) and TRITC phalloidin (for actin F). The images were captured with a CCD camera (Axiocam MRM) adapted to a Zeiss Axio Observer D1 inverted microscope and analysed using Axiovision Release 4.6.3. software. The software was calibrated to measure actual dimensions for the 10× objective lens using a stage micrometer. Adherent cells were observed on the surface of plastic dishes (negative control) and on all titanium implants in a similar number. Positive control consisting by resins compound discs did not allow cells attachment (Figure 3).

**Figure 4:**
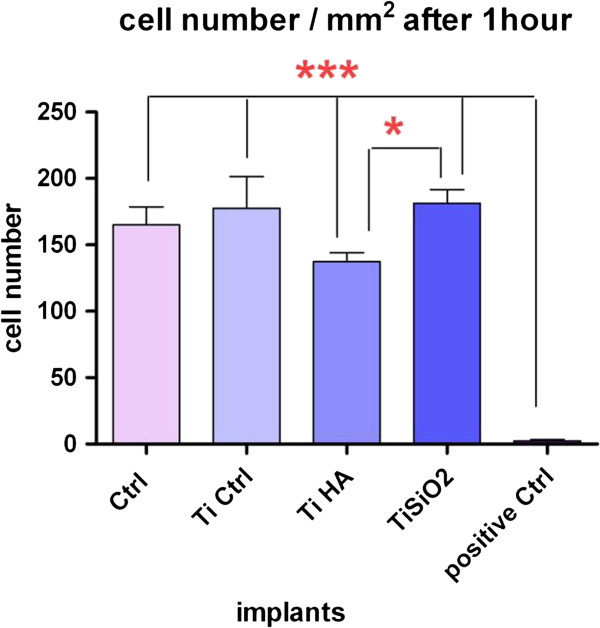
**Cell count results one hour after seeding of the osteoblasts on the implants.** Legend: cells cultivated on plastic dishes-Negative Ctrl, noninfiltrated, control Ti (Ti control), titanium infiltrated with hydroxyapatite (Ti HA), Ti infiltrated with silicatitanate (Ti SiO_2_) and composite resins as positive Ctrl.

**Figure 5:**
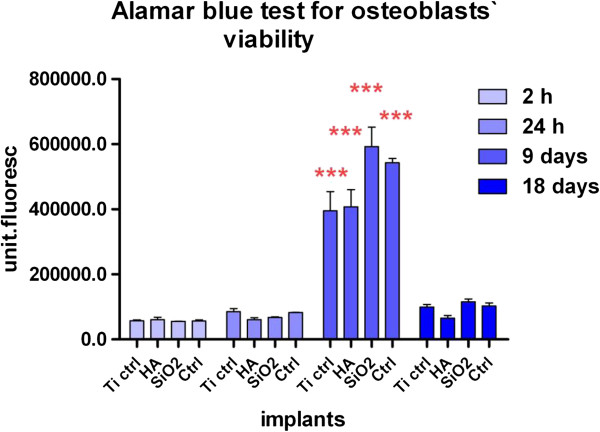
**The viability of the osteoblasts on the titanium implants, evaluated with Alamar blue assay and showing their adhesion to the substrates (at 2 h) and their proliferative capacity (on day 1, 9 and 18 day).** Legend: noninfiltrated, control Ti (Ti control), titanium infiltrated with hydroxyapatite (Ti HA), Ti infiltrated with silicatitanate (Ti SiO_2_), cells cultivated on plastic dishes (ctrl ) ***p < 0.0001.

Cells were counted in 3 different microscopic fields, through random selection. Using the unpaired t test with Welch’s correction we found statistically significant differences between the Ti Ctrl and the other implants (Ti HA, Ti SiO_2_ and positive control) with the first more intensely promoting cell attachment in the first hour (Figure 4).Osteoblast adhesion at two hours and their proliferation at 24 hours, 9 days and 18 days were evaluated using the Alamar blue assay. The results are illustrated in Figure 5 and they show a similar ability of all implants to induce cell adhesion early after seeding.

The differences between the Alamar blue test results and cell count results can be explained by the fact that Alamar blue test incorporates a fluorometric/colorimetric REDOX indicator based on detection of innate cellular metabolic activity. Another possible explanation is that the cells were counted at 1 hour after seeding and the viability test was performed at 2 hours, and this period of time was probably decisive for the completion of cell adhesion on Ti HA implants. In some experimental models it was shown that certain reductases involved in the reduction of Alamar blue are present not only in mitochondria, but in subcellular components and Alamar blue reduction may signify an impairment of cellular metabolism [35]. Some interactions and assay compatibility problems have been specifically demonstrated in screening bioengineered nanomaterials [36]. Another possible explanation is the integrin-triggered production of reactive oxygen species (ROS) derived from mitochondria by signals generated during the early phase of cell attachment with associated changes in the cytoskeleton and in the phosphorylation levels of several proteins [37].

**Figure 6:**
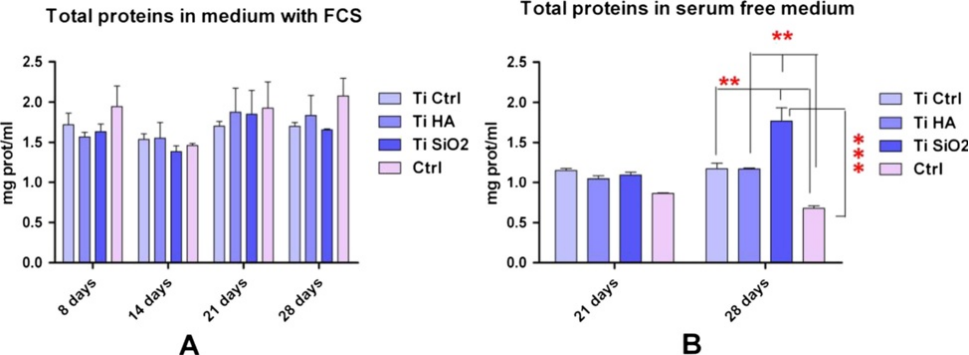
**Levels of total proteins secreted in culture mediums by osteoblasts cultivated on the implants’ surface, measured with the Bio-Rad protein microplate assay. A**. in complete medium with fetal calf serum, FCS (measured on day 8, 14, 21 and 28); **B**. in serum-free medium (measured on day 21 and 28) Legend: noninfiltrated, control Ti (Ti control), titanium infiltrated with hydroxyapatite (Ti HA), Ti infiltrated with silicatitanate (Ti SiO_2_), cells cultivated on plastic dishes (ctrl).

**Figure 7:**
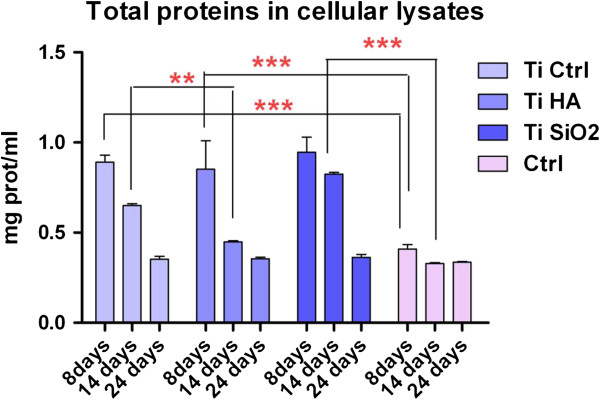
Levels of total proteins secreted in cellular lysates of osteoblasts cultivated on the implants and collected on day 8, 14 and 24, as measured with the Bio-Rad protein microplate assay (***p < 0.001; **p < 0.01). Legend: noninfiltrated, control Ti (Ti control), titanium infiltrated with hydroxyapatite (Ti HA), Ti infiltrated with silicatitanate (Ti SiO_2_), cells cultivated on plastic dishes (ctrl).

In our experiments, the statistical analysis with Two-way ANOVA Bonferroni posttests showed a significant increase of sample fluorescence intensity for all Ti implants and controls cells without substrate after 9 days of cultivation, with a significant advantage for Ti SiO2 implants. Cell viability decreased dramatically after 18 days, as a possible consequence of the progression of the cells to a more advanced stage of differentiation.The effect of various substrates and media formulation on cell growth and proliferation was determined by the assessment of the rate of total protein synthesis using the microplate BioRad Protein test. The results are displayed in Figure 6.In the presence of fetal serum, no significant differences between samples were observed (Figure 6A). Differences emerged when cells were cultivated in serum-free conditions, especially for cells grown on Ti SiO2 implants (Figure 6B). Statistical analysis with two-way ANOVA Bonferroni posttest showed significant differences at 28 days, favoring the implants of Ti infiltrated with SiO2 (***p < 0.001).It is known that cellular lysates should reflect the amount of total intracellular proteins. Protein levels were determined in the cellular lysates using the same microplate BioRad Protein assay. We found that the protein levels in the cellular lysates decreased progressively in time in all Ti samples (Figure 7).

This finding suggests a decreased synthesis of proteins when cells progress in more advanced stage of differentiation, a decrease in cell number (as observed with Alamar test), or both.

### Osseoconductivity and osseointegration: osteoblast differentiation and *de novo* mineralization

**Figure 8:**
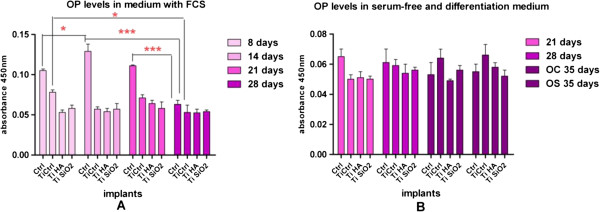
**Levels of osteopontin (OP) secreted in the culture medium by osteoblasts cultured on the implants’ surface. A**. in medium wih fetal calf serum (FCS), collected on day 8, 14, 21 and 28 (analysis with two-way ANOVA Bonferroni posttest *p < 0.05; **p < 0.01; ***p < 0.001); **B**. in serum-free medium (collected on day 21 and 28), in simple (OS) and in complex (OC) differentiation mediums (collected on day 35). Legend: cells cultivated in plastic dishes (ctrl), non-infiltrated, control Ti (Ti control), titanium infiltrated with hydroxyapatite (Ti HA), Ti infiltrated with silicatitanate (Ti SiO_2_).

**Figure 9:**
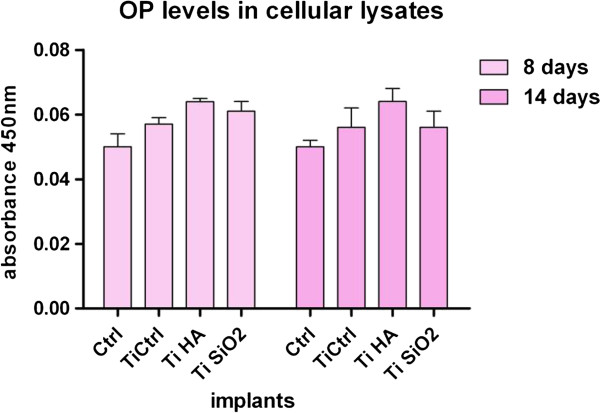
**Levels of osteopontin (OP) in cellular lysates of osteoblasts cultured on the titanium implants and measured on day 8 and 14 of cultivation.** Legend: non-infiltrated, control Ti (Ti control), titanium infiltrated with hydroxyapatite (Ti HA), Ti infiltrated with silicatitanate (Ti SiO_2_), cells cultivated on plastic dishes (ctrl).

In the present study, OP was determined through an ELISA method both in cell culture medium (complete and serum-free mediums) and in cellular lysate. The evaluations were made in samples harvested at day 8, 14, 21 and 28 in the case of medium with FCS and at day 21 and 28 for serum-free medium. Cell lysates were harvested after 8, 14 and 24 days of cultivation (Figure 8). Medium collected from osteoblasts cultured on plastic dishes was used as for comparison.One interesting finding is that of consistently higher values of the OP in samples grown on plastic in the presence of FCS (Figure 8A). One explanation might involve the immobilization of the OP protein in the newly synthesized matrix on Ti implants. We also found differences in OP secretion depending on culture conditions: in simple osteogenic medium, OS (containing 10 nM dexamethasone, 50 μg/ml ascorbic acid and 10 mM β-glycero-phosphate) or in complex osteogenic medium, OC (supplemented with growth factors: 3 ng/ml TGFβ1 and 4 ng/ml BMP2) (all reagents acquired from Sigma Aldrich). These differentiation-favorable environments induced an increase in the OP level for the Ti Ctrl implants (Figure 8B).The assessment of OP levels in cell lysates, at 8 and 14 days of culture, showed no differences between substrates (Figure 9).

**Figure 10:**
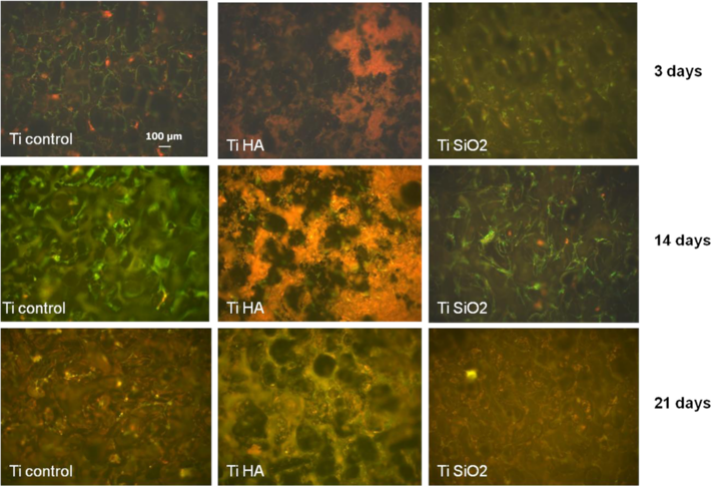
**Immunocytochemical staining of the osteoblasts cultivated on the surface of the implants showing the expression of osteopontin (OP).** Osteoblasts were stained with an anti OP-FITC monoclonal antibody on day 3, 14 and 21 days (magnification 100×). Legend: non-infiltrated control Ti (Ti control), titanium infiltrated with hydroxyapatite (Ti HA), Ti infiltrated with silicatitanate (Ti SiO_2_).

**Figure 11:**
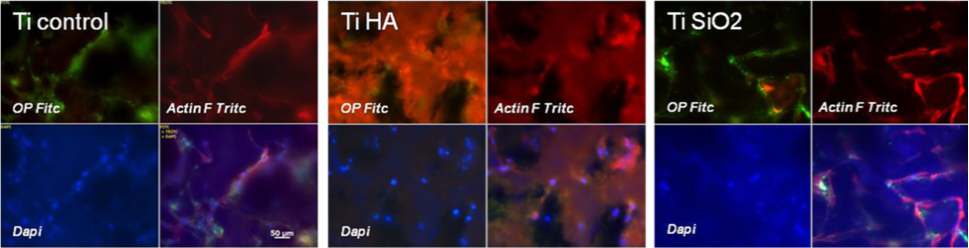
**Immunocytochemical staining of osteoblasts on the surface of the implants (noninfiltrated Ti, Ti infiltrated with HA and Ti infiltrated with SiO**_**2**_**) showing the expression of actin F and osteopontin (OP).** Osteoblasts were stained with anti OP-FITC monoclonal antibody, phalloidin TRITC (for actin F) and DAPI (for nuclei). The images were taken on day 14 and composed with Axiovision Release 4.6.3. image analysis software (magnification 200×). Legend: non-infiltrated, control Ti (Ti control), titanium infiltrated with hydroxyapatite (Ti HA), Ti infiltrated with silicatitanate (Ti SiO_2_).

The expression of OP was also studied in immunostained samples fixed after 3, 14 and 21 days. Each types of implant (Ti Ctrl, Ti HA and Ti SiO_2_) showed positivity for OP until day 14, after which the protein expression decreased (Figure 10). This phenomenon can be explained by an earlier initiation of the mineralization process (especially for implants with HA), which could be correlated with the disappearance of OP, as some authors consider OP as an inhibitor of mineralization [38,39].For a better visualization of the expression of OP and of cell adhesion to the implants’ surface, we used a triple staining with fluorescein isothiocyanate (FITC) for osteopontin expression, phalliodin TRITC for F-actin fibers and DAPI for nuclei (Figure 11).

This triple coloration allowed a more accurate observation of osteoblasts cultured for 14 days on titanium implants. DAPI staining of nuclei showed the number per microscopic field of cells adhered to the implants’ surface, with no notable differences between implants. OP protein stained with FITC-conjugated antibodies, showed characteristic intracellular aggregates with increased fluorescence intensity in cells cultivated on Ti Ctrl and Ti SiO_2_ implants. Cells grown on the HA implants were probably embedded and masked by HA crystals. Actin filaments (stained with TRITC phalloidin) had a more intensive and obvious expression in the case of control Ti and SiO_2_ Ti implants, as these substrates promoted the adherence of osteoblasts on a larger area while also inducing a flattened shape and the development of dendritic spines. Cells grown on Ti HA had a more compressed cell body with extensions that could not be seen due to the HA layer. In our experiments only Ti SiO_2_ implants showed expression of stress fibers.

**Osseointegration** is a later stage in the evolution of an implant and is characterized by the establishment of bone-implant contact and the development of peri-implant bone as a consequence of osteogenic mineralization [40]. In the present study, mineralization was assessed by quantifying calcium deposition on Ti implants with a modified Alizarin red method. Evaluations were made in Ti implants seeded with osteoblasts, after 14, 21 and 28 days of cultivation in medium with FCS or serum-free medium. Because Ti HA implants contain large amounts of calcium, we assessed the amount of calcium in Ti HA implants without cells growing on their surface and the results were subtracted from those obtained for Ti HA implants cultivated with cells.

**Figure 12:**
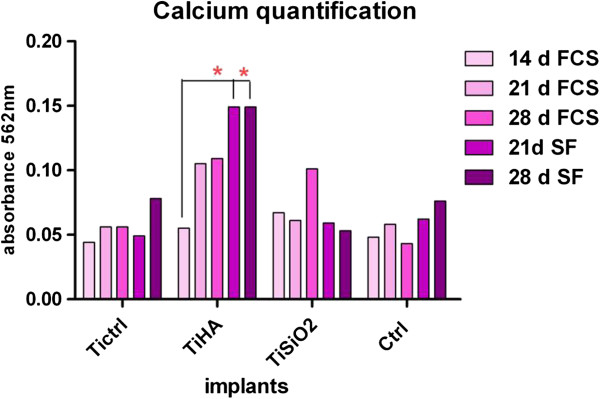
**Levels of calcium deposition on the implants by osteoblasts cultured in medium with fetal calf serum, FCS (measured on day 14, 21 and 28) and in serum-free medium, SF (measured on day 21 and 28).** Quantification of the Calcium content was done using a modified Alizarin red method. Legend: noninfiltrated, control Ti (Ti control), titanium infiltrated with hydroxyapatite (Ti HA), Ti infiltrated with silicatitanate (Ti SiO_2_), cells cultivated on plastic dishes (ctrl).

In the first 14 days an equal amount of calcium was observed in all titanium implants, when cultivated in complete medium. After 21 and 28 days of cultivation in complete medium, a gradual increase in calcium deposits occurred mostly in Ti HA implants and, in a lower degree, in Ti SiO_2_ implants. Serum-free medium significantly increased the deposition of calcium on the surface of Ti HA implants and on control samples after 28 days of cultivation (Figure 12).

**Figure 13:**
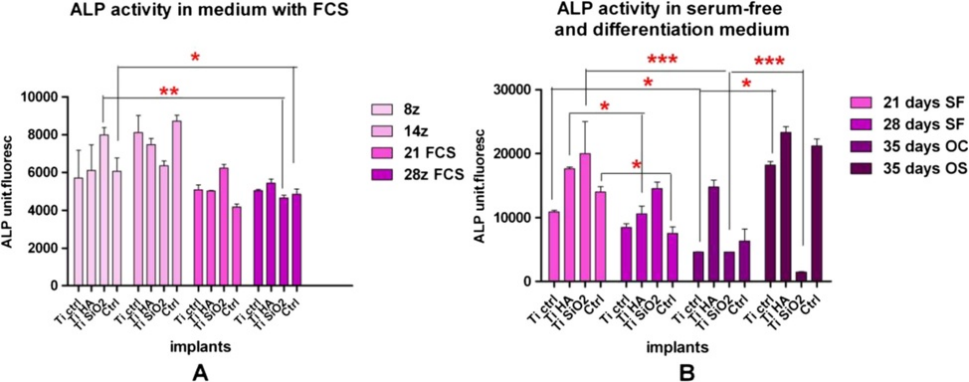
**Levels of alkaline phosphatase activity in the mediums containing osteoblasts cultured on implant surfaces. A**. in medium wih fetal calf serum (FCS), evaluated on day 8, 14, 21 and 28 (*p < 0.05); **B**. in serum-free medium, SF (collected on day 21 and 28), simple (OS) and complex (OC) differentiation mediums (collected on day 35) Legend: noninfiltrated, control Ti (Ti control), titanium infiltrated with hydroxyapatite (Ti HA), Ti infiltrated with silicatitanate (Ti SiO_2_), cells cultivated on plastic dishes (ctrl).

Evaluation of alkaline phosphatase (ALP) activity in the environment of osteoblasts cultured on Ti implants revealed different cell behaviors depending on the type of cultivation medium (medium with FCS, serum-free medium, simple osteogenic and complex osteogenic differentiation medium), culture duration and the type of titanium implants (Figure 13). The highest levels of ALP activity were obtained in Ti Ctrl implants and controls without substrate after 14 days of cultivation in complete medium and for Ti SiO_2_ implants after 21 days (Figure 13A). (Two-way ANOVA Bonferroni posttest; **p < 0.01). A common trend of progressive decline in ALP activity was observed after 28 days. In serum-free conditions ALP activity increased significantly after 21 days in Ti SiO_2_ implants, and after 35 days in Ti Ctrl and Ti HA implants as well control samples in simple osteogenic medium (Figure 13B). (Two-way ANOVA Bonferroni posttest; *p < 0.05; **p < 0.01).

**Figure 14:**
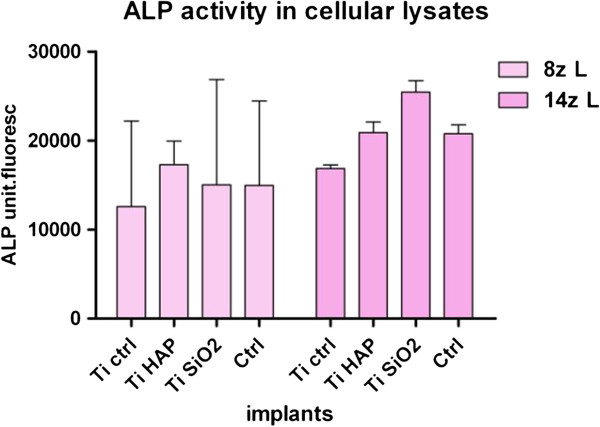
**Levels of alkaline phosphatase activity in cellular lysates from osteoblasts cultured on titanium implants in complete medium with fetal calf seum, FCS.** Evaluation on day 8 and 14. Legend: noninfiltrated, control Ti (Ti control), titanium infiltrated with hydroxyapatite (Ti HA), Ti infiltrated with silicatitanate(Ti SiO_2_), cells cultivated on plastic dihes (ctrl).

ALP activity was also evaluated in cellular lysates obtained after 8 and 14 days of osteoblast cultivation. The results showed higher levels of the enzyme in cellular lysates when compared with the complete medium-derived samples. At 14 days, the highest ALP activity was found in the lysates obtained from the cells cultivated on Ti SiO_2_ implants (Figure 14).

### Collagen detection

**Figure 15:**
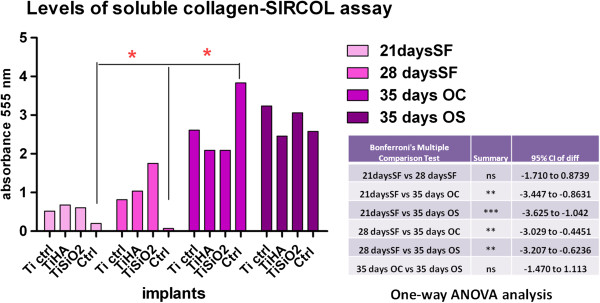
**Levels of soluble collagen produced by the osteoblasts cultured on titanium implants in serum-free medium (collected on day 21 and 28), simple (OS) and complex (OC) differentiation mediums (collected on day 35).** Legend: noninfiltrated, control Ti (Ti control), titanium infiltrated with hydroxyapatite (Ti HA), Ti infiltrated with silicatitanate (Ti SiO_2_), cells cultivated on plastic dihes (ctrl) Table: One-way ANOVA analysis reveals statistical differences between the medium of cultivation (SF vs. OS and OC serum-free medium).

Levels of the most abundant protein of the extracellular matrix, collagen, were determined with the SIRCOL collagen assay, in samples cultured in serum-free medium (SF), simple osteogenic medium (OS) and complex osteogenic differentiation medium (OC). Medium was harvested on day 21 and 28 (for the serum-free medium) and on day 35 (for the two differentiation mediums). In the serum free medium, an important increase in soluble collagen concentration was seen on day 28 (compared with day 21) for the Ti SiO_2_ implants (Figure 15). The results also showed significant differences between culture mediums with respect to their content in soluble collagen, as one-way ANOVA tests showed. Notable differences were observed between all samples of Ti implants and control without substrate cultivated in medium with FCS and samples cultivated 28 and 35 days in simple osteogenic medium (OS) and complex osteogenic medium (OC). Analysis with two-way ANOVA Bonferroni posttest; *p < 0.05 showed a significant increase of soluble collagen in control samples after 35 days of cultivation in OC medium (Figure 15).

**Figure 16:**
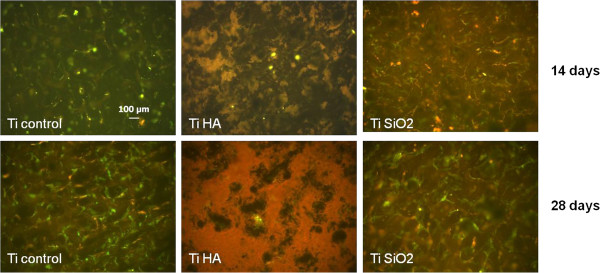
**Immunocytochemical staining (FITC) for collagen 1A of the osteoblasts cultured in complete medium on titanium implants and evaluated on day 14 and 28.** Legend: noninfiltrated control Ti (Ti control), titanium infiltrated with hydroxyapatite (Ti HA), Ti infiltrated with silicatitanate (Ti SiO_2_) (magnification 100×).

Collagen secretion was also investigated by immunocytochemical staining of cells grown on titanium samples. Type I collagen was expressed by all samples grown on titanium, with a higher observed intensity in control Ti and Ti infiltrated with SiO_2_ after 14 days and much more intensely after 28 days of cultivation (Figure 16).

**Figure 17:**
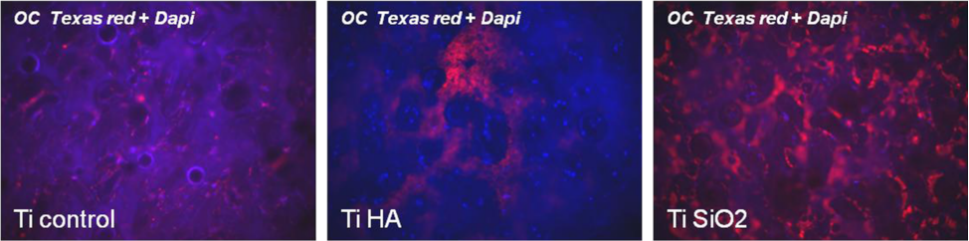
**Immunocytochemical staining for osteocalcin Texas red, counterstained with DAPI in osteoblasts cultured in complete medium on titanium implants evaluated after 28 days of cultivation.** Legend: non-infiltrated, control Ti (Ti control), titanium infiltrated with hydroxyapatite (Ti HA), Ti infiltrated with silicatitanate (Ti SiO_2_) (magnification 100×).

The osteoblast-osteocyte transition was observed after 28 days of cultivation especially on titanium samples with SiO_2_ and in a lower degree on control titanium-cultured samples. This was suggested by the strong expression of osteocalcin, as revealed through immunocytochemical staining. In silicatitanate- and HA-infiltrated implants, deposition of osteocalcin seems to occur in the extracellular matrix, with a more uniform distribution of the protein observed in the case of SiO_2_ implants. In comparison, in control titanium implants osteocalcin was located in the intracellular space of the osteoblasts (Figure 17).

**Figure 18:**
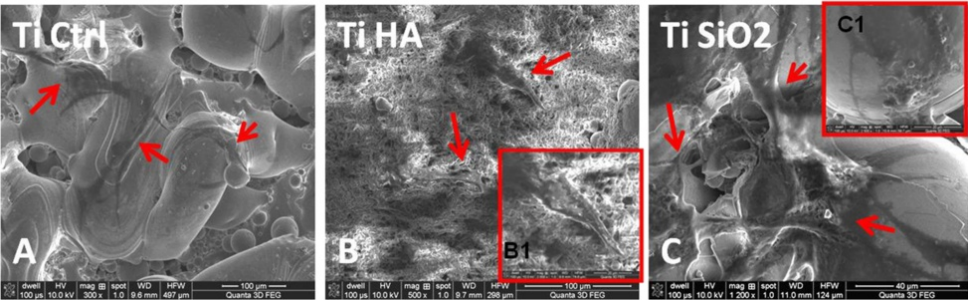
**Scanning electron microscopy images of osteocytes attached to the surface of titanium implants after 28 days of cultivation.** Legend: Osteocytes are indicated by arrows. **A**. noninfiltrated control Ti (magnification ×300); **B**. Ti infiltrated with hydroxyapatite (Ti HA) (magnification ×500); B1- image of an osteoblast with 2000× magnification; **C**. Ti infiltrated with silicatitanate (TiSiO_2_) (magnification ×1200); C1- image of an osteocyte with ×2500 magnification.

SEM images taken after 28 days show various obvious morphological changes of cells depending on the type of implant. A typical osteocytic morphology - with long dendrite-like processes interacting with neighboring cells - was observed mainly on the SiO_2_ surface implant and to a lesser extent in control titanium (Figure 18A and C). Ti HA implants had a more particular morphology: the cells appeared to be partially included in the HA and in the newly formed matrix substrate. The cell extensions were also likely to be masked (Figure 18B).

## Discussions

Osseoinduction, osteoconduction and osseointegration are the three interdependent steps involved in bone healing, and the specific response of the bone to implant insertion is similar to its response to fractures [41]*.* O*sseoinduction* – defined by Friedenstein in 1968 as the recruitment of undifferentiated osteoprogenitor cells and their induction into osteogenic competent cells - depends mainly on cell adhesion phenomena [42,36]. The quality of the interface between cells and implants is essential for the induction of events involved in implant integration [43]. The adhesiveness of bone grafts/implants is related to their ability to support selective attachment, proliferation, differentiation and migration of anchorage-dependent cells [44]. Some examples of osseoinductive materials used as implants are: the polymer polyhydroxyethylmethacrylate (poly-HEMA), metals (porous titanium), natural materials such as hydroxyapatite (HA), synthetic ceramics and composites such as HA/poly(lactic-coglycolic acid) (PLGA) or HA/collagen [2].

The aim of this study was to compare the osseoinductive, osteoconductive and osseointegrative properties of two kinds of titanium implant coatings, with the purpose of selecting materials with increased bioactivity. Ti6Al7Nb implants with 25% total porosity, processed with SLM technology, were infiltrated with hydroxyapatite and silicatitanate through a sol–gel method. Gravimetric method revealed a slow decrease of porosity to 22% for Ti HA implants and to 23% for Ti SiO_2._ Uncoated Ti implants were used as controls. SEM and EDX analysis revealed that the two methods of coating conferred, as expected, different physical and chemical properties. For the Ti Ha implants, the EDAX analysis resulted in 30.25% Ti content (A%) with a Ca/P ratio of 2.3. The attachment of cells on the Ti HA scaffolds was delayed in the first hour when compared to Ti SiO_2_ and porous uncoated Ti implants, as revealed by SEM images and cell counting. However, this initial disadvantage was subsequently surmounted, as evidenced by the assessment of proliferation with Alamar blue and total protein content. The metabolic activity of osteoblasts was highest after 8–9 days of cultivation in all Ti implants, with an observed advantage in SiO_2_ infiltrated Ti implants. This advantage was maintained until day 28, as proved by the increasing protein content in serum-free medium. Similar results were obtained by Waselau et al. [45] in a comparative study analyzing the effects of bioactive glass S53P4 and beta-tricalcium phosphate on osteogenic differentiation of human adipose stem cells in the presence of BMP-2 and BMP-7. qDNA measurements revealed significantly greater cell populations in the case of bioactive glass used with control medium after 7 days and in osteogenic medium after 14 days [45].

The HA coatings are biomimetic and many studies proved their osteoconductivity. On the other hand, their osseoinductive ability is more controversial and still highly debated. This may be due to differences in the manufacturing process, which result – even in case of equal chemical compositions – in different levels of microporosity and surface roughness, which are essential factors that determine the osseoinductive potential of the material [42,36].

The silicatitanate gel used for Ti coating in our experiments contained 6.99% silica and demonstrated a good osteoinductive activity, sustaining cell adhesion and proliferation. The sol–gel method used for obtaining this coating is now preferred to the classical melting method because of its advantages [46]. It is a much more flexible method for obtaining amorphous multi-component oxide systems of high purity and homogeneity, at lower temperatures, with a controlled porosity [47]. By forming nanopores in the glass, the surface area becomes enlarged and provides sites for cell attachment [48,49]. Also, the hydroxyl groups located in the sol–gel of bioactive glasses structures confer a higher bioactivity when compared to the bioactive glasses prepared by melting techniques. In addition, by releasing soluble silica and calcium ions, it promotes differentiation of osteoprogenitor cells [50,51]. Many *in vitro* studies investigated the role of silicon (Si) in bone homeostasis and reported that Si increased the synthesis of non-collagenous matrix polysaccharides and of collagen, due to increased prolyl-hydroxylase activity (the enzyme involved in collagen synthesis). Si also exerts a direct influence on osteoblasts, promoting cell proliferation, extracellular matrix synthesis, alkaline phosphatase (ALP) activity and osteocalcin synthesis [52].

*Osteoconductive materials* allow bone growth on the surface of materials and into their pores, channels or pipes, which also depends on previous osseoinduction and on proper blood supply. Signals from cells adhered to the biomaterial’s surface trigger the secretion of many growth factors with mitogenic and angiogenic activity, such as: insulin-like growth factors I and II (IGF I, II), fibroblast growth factor (FGF), transforming growth factor (TGF-β), vascular endothelial growth factor (VEGF) and platelet-derived growth factor (PDGF), as well as the secretion of bone tissue-related growth factors with osteogenic potential: bone morphogenic proteins 2 and 7 (BMP-2 and BMP-7) [3,45]. *Osseointegration* allows bone anchorage and consolidation between the newly formed bone and the implant. Because initial osseointegration is dependent on bone induction and conduction, materials that are too toxic to allow cellular adhesion and development into bone forming cells will not be osseointegrated [3]. Formation of hydroxyapatite and surrounding bone tissue by binding to the extracellular matrix is essential for osseointegration. The most important questions to answer when investigating a system of osteoblasts cultured on implants are whether the substrate supports de novo matrix mineralization, if it is similar to its naturally occurring counterpart, and how it could be distinguished from the presence of minerals contained in the substrate.

Osteoconductivity and osseointegration of Ti HA, Ti SiO_2_ and porous Ti implants used in the present study were evaluated by assessing the differentiation and mineralization processes. Osteopontin (OP) is one of the most important non-collagenous phosphoproteins and an indicator of the differentiation process of osteoblasts. OP expression was evaluated in different culture media (complete medium, serum-free medium and osteogenic medium) at different points in time. Using the ELISA method, no significant differences were found among the implants. Interestingly, the highest OP values were detected on day 14, for the cultures grown in complete medium on plastic dishes. This may be explained by the immobilization of OP on the implant surface in the newly synthesized matrix, as OP was reported to be present both in an immobilized form in the extracellular matrix of mineralized tissues and in a soluble form in tissue fluids [53,54]. The osteogenic differentiation medium induced a discrete increase in OP levels after 35 days of osteoblast cultivation on control Ti implants. A strong OP expression, evaluated by immunocytochemical staining, was detected after day 3 for Ti Ctrl and Ti SiO_2_ implants. It continued to increase until day 14 and decreased after 21 days. With a combined immunostaining for osteopontin and filamentous actin, different behaviors of implants were observed with regard to the rearrangement of actin fibres. The Ti SiO_2_ and Ti Ctrl implants induced a more flattened morphology of cells, and were associated with the expression of stress fibers.

The actin cytoskeleton is involved in cell adhesion and cell motility, and its fiber arrangement modulates cell shape differently depending on the type of nanoscale and microscale surface roughness as well as the patterning of the surface of the material on which cells are grown. Mechanical stimulation of osteoblasts or stem cells induced by the properties of the implant surface triggers the reorganization of the focal adhesion plaques followed by the rearrangement of the cytoskeleton and the activation of signaling pathways involved in osteogenic cell differentiation such as transcription factors Cbfa1 (Core Binding Factor A1) and Osterix. As a consequence osteoblasts synthesize higher amounts of collagen I, osteopontin, osteocalcin and bone sialoprotein, and induce higher levels of alkaline phosphatase activity [55-57]. The canonical Wnt (Wingless/Integrated) signaling pathway is also activated with consequences on β-catenin, alkaline phosphatase and osteocalcin expression, as Galli et al. showed in a study using mesenchymal and osteoblastic cells growing on polished titanium discs versus acid-etched and sand-blasted (SLA) surfaces. Differences were also observed among various cell types [58]. For instance, OP is also involved in cell adhesion phenomena of cell-cell or cell-extracellular matrix interactions that occur during cell proliferation and migration. Elevated concentrations of osteopontin were found in sites of bone resorption and its expression was strongly increased by mechanical stimuli. The effects of OP on HA formation seem to be related to its phosphorylation state. Exogenous phosphorylated OP is known to inhibit mineralization, but dephosphorylation by tissue-nonspecific alkaline phosphatase (ALP) reverses this effect [38,39]. The phosphorylation state of OP can be influenced by the physical and chemical properties of the substrate and, as a consequence, modulates its signaling ability [59]. This fact may explain the different cell responses observed in our experiments, most notably the increased expression of OP in complete medium after 8–14 days especially in SiO_2_ Ti and control Ti implants, which correlates with concomitant increases of other specific proteins such as ALP.

**Figure 19:**
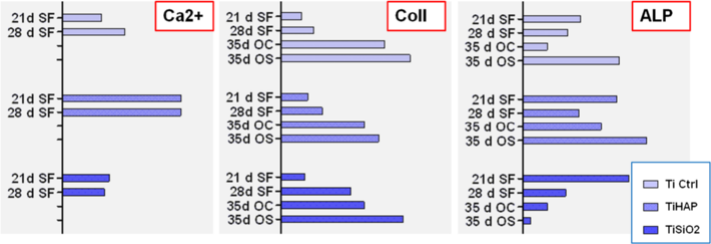
**A schematic synthesis of results regarding the most important elements implicated in the mineralization process.** Ca^2+^ deposition, collagen synthesis and ALP activity, in samples cultivated in serum-free medium (SF) and in differentiation medium (complex osteogenic medium OC and simple osteogenic medium OS) Legend: noninfiltrated, control Ti (Ti control), titanium infiltrated with hydroxyapatite (Ti HA), Ti infiltrated with silicatitanate (Ti SiO_2_).

ALP is a key enzyme that can both promote and inhibit mineralization. It is a cell-membrane-associated enzyme, expressed also in matrix vesicles, and in association with other proteins enhances deposition of hydroxyapatite along the collagen fibrils. ALP hydrolyzes its substrate, inorganic pyrophosphate, to inorganic phosphate, the latter being a substrate for the HA mineral [60]. In our experiments, the highest activity of ALP was found in the samples obtained from the lysate of cells cultivated on Ti SiO_2_ implants. However, the activity of ALP was much higher (2–2.5 fold) in serum-free conditions, especially in Ti HA and SiO_2_ implants. ALP activity decreased rapidly after 28 days, indicating that the mineralization process is nearly complete. In osteogenic differentiation mediums, the expression profile of ALP was different. A strong ALP activity was detected on day 35 in simple osteogenic medium from the Ti Ctrl and Ti HA, accompanied by elevated levels of collagen. SiO_2_ implants did not respond to the differentiation medium in terms of ALP activity, but exhibited similar levels of collagen. The presence of BMP-2 and TGF β1 in the complex osteogenic medium increased ALP activity only in Ti HA implant cultures (Figure 19).

Collagen synthesis, as an indicator of matrix formation, was significantly increased in Ti SiO_2_ samples after 28 days. In addition, higher soluble collagen levels were observed in control Ti and Ti SiO_2_ implants cultivated in simple osteogenic differentiation medium. These findings do not exclude possibly similar collagen synthesis on Ti HA implants, yet we could not demonstrate this because in such implants the collagen fibers could not be visualized (as they may have been masked by the accelerated deposition of newly formed HA crystals).

Calcium deposition was investigated as a marker of mineralization. Differences between implants emerged after 21 and 28 days of cultivation in complete medium, with a gradual increase in calcium deposition observed mostly in Ti HA implants and in a lower degree in Ti SiO_2_ implants. Serum-free medium significantly increased the deposition of calcium on the surface of Ti HA implants after 28 days of cultivation. A similar outcome was reported by Yamada et al. [61], as they observed accelerated bone-implant integration after the addition of pure nanopolymorphic crystalline HA to micro-roughened titanium, as well as a significantly increased osteoconductivity [61].

The complex osteogenic differentiation medium used in this study also contained TGF-β1 (in a low dose-3 ng/ml), a growth factor with dual biological activity, as it can regulate osteoblast differentiation not only positively but also negatively. Ochiai et al. [62] observed that only repeated high doses of TGF-β1 suppressed osteoblast differentiation with decreased ALP activity, although a unique low dose of TGF-β1 strongly induced osteoblast differentiation [62]. On the other hand, TGF-β1 protects pre-osteocytes from apoptosis [63]. Similar results regarding the osteogenic medium were obtained by Tirkkonen et al. [64] in an adipose stem cell osteogenic differentiation study that compared the efficiency of BMP-2 and BMP-7, VEGF and osteogenic medium. Stem cells were grown on commercially available bioactive glass scaffolds and biphasic granules. The bioactive glass induced an increased cell proliferation whereas calcium phosphate led to a more significant collagen production. They did not find a benefit after the addition ofgrowth factors in comparison with plain osteogenic medium. Moreover, BMP-7 inhibited the proliferation and osteogenic differentiation of adipose stem cells [64]. The lack of improvement of the osteogenic differentiation of stem cells through the addition of BMP-2 or BMP-6 was also reported by other authors [65-67]. The master of controlling osteoblast differentiation into osteocytes is RUNX2 (runt-related transcription factor) [68] and in this context BMP2 induces osteoblast differentiation through Runx2-dependent ATF6 (bZIP transcription factor) expression, which directly regulates osteocalcin transcription [69]. Prior to the mineralization process, as the cells pass through progressive differentiation stages, some specific markers are revealed, the earliest being RUNX2, followed by ALP (an early-mid marker), osteopontin (OP), osteocalcin (OC) and osteonectin (ON) [70]. Roach (1996) studied the roles of matrix non-collagenous proteins such as OP, OC, bone sialo-protein (BSP) and ON and their tissue localization using double immuno-histochemistry. The appearance of OP and BSP ahead of the mineralization front, prior to mineralization, suggested that both proteins are necessary for the initiation of bone mineralization. OC and ON were present in fully mineralized matrix, possibly having a function in controlling the amount and rate of crystals formation [71].

In our study, after the immunocytochemical analysis, osteocalcin had the highest expression in Ti SiO_2_ and Ctrl Ti implants after 28 days of cultivation, and it was associated with increased expression of collagen I. Such effects of bioactive glass were explained by Silver et al. [72], through a marked alkalinization of the intracellular and extracellular environment that influence the activities of intracellular enzymes and signaling pathways, which in turn increases collagen synthesis [72]. Similar observations were reported regarding orthosilicic acid or different types of bioglasses [73,74]. Osteocalcin, a terminal marker of osteoblastic differentiation, is present in high amounts in the bone matrix. It binds calcium and inhibits bone growth by inhibiting the activity of transglutaminase [75]. After posttranslational modification with the addition of carboxylated glutamic acid residues (gla residues), OC gains a high affinity for hydroxyapatite crystals. The inhibitory role of OC possibly involves a conservation of cells in a late-osteoblast stage of differentiation, with associated prevention of osteocytic differentiation [76] and initiation of the remodeling process of new bone.

Through interpretation of the results derived from our study, two kinds of osseointegrations were found. The first type consists of a predominant collagen protein matrix construct and a self-limiting mineralization process through increased expression of osteocalcin, which was observed in the case of the porous titanium and infiltrated silicatitanate implants. The second type belongs to HA-infiltrated titanium, characterized by an early increase in calcium deposits, with a lesser degree of collagen and non-collagenous protein synthesis. Both types of osseointegration have potential applications, however *in vivo* studies are required to clarify the most suitable type of implant depending on the site of implantation (considering the mechanical load and the type of bone in need of repair) as well as the associated pathological conditions such as osteoporosis. Despite some limitations regarding the mechanical properties of porous apatite ceramics, the excellent osteoconductivity of HA coatings and the possibility of associating bioactive molecules (such as proteins, aminoacids, antibiotics, growth factors, anticancer and anti-osteoporosis drugs) to calcium phosphate indicate the potential advantages of the use of this type of coating especially in osteopenic bone repair [77,78]. On the other hand, the osseoinductive properties of SiO_2_-coated Ti implants (as described in our study) and their ability to promote angiogenesis and enhance neocartilage formation [73], can provide other important applications in implantology, especially for repairing bones at sites where mechanical strength is of lesser importance.

## Conclusions

Two types of titanium coatings were comparatively studied with regard to bone regeneration, and they induced different behaviors of osteoblastic cells. Ti implants infiltrated with hydroxyapatite (HA) demonstrated an increased capacity to induce early mineralization with a lower ability to induce cell adhesion and proliferation. Conversely, the implants infiltrated with SiO_2_, as well as porous titanium preserved the attachment and adhesion of osteoblasts, promoted celldifferentiation and induced the production of the protein component of the extracellular matrix (collagen and the non-collagenic proteins osteopontin and osteocalcin). The addition of growth factors BMP-2 and TGFβ1 in the differentiation medium did not improve the mineralization process. Both types of coatings have their advantages and limitations, which can be exploited depending on the local conditions and on the bone lesions in need of repair. Their properties can be improved through methods of functionalization with biomolecules involved in osteogenesis.

## Competing interests

The authors declare that they have no competing interests.

## Authors’ contributions

Study design: PB, SS, CP, IB, OS, VC Scaffolds manufacturing: CP, MT, SS, CB Scanning electron microscopy and EDX analysis: AV Cell cultures: MPS, ND, OB Biocompatibility assays: OS, GC, PV, CB Data analysis: IB, OS, TM, AV, CP, MT Data interpretation. IB, OS, Manuscript preparation: IB, OS, MPS, MT, AV. Approving final version of manuscript: IB and OS takes responsibility for the integrity of the data analysis. All authors read and approved the final manuscript.
